# Whole Genome Sequencing of Greater Amberjack (*Seriola dumerili*) for SNP Identification on Aligned Scaffolds and Genome Structural Variation Analysis Using Parallel Resequencing

**DOI:** 10.1155/2018/7984292

**Published:** 2018-03-28

**Authors:** Kazuo Araki, Jun-ya Aokic, Junya Kawase, Kazuhisa Hamada, Akiyuki Ozaki, Hiroshi Fujimoto, Ikki Yamamoto, Hironori Usuki

**Affiliations:** ^1^Research Center for Aquatic Breeding, National Research Institute of Aquaculture, Fisheries Research Agency, 224 Hiruda, Tamaki-cho, Watarai, Mie 519-0423, Japan; ^2^Marine Biological Science, Faculty of Bio-resources, Mie University Graduate School, 1577 Kurimamachiya-cho, Tsu City, Mie 514-8507, Japan; ^3^Marine Farm Laboratory Limited Company, 309 Takahiro Tachibaura Otsuki-cho, Hata-gun, Kochi 788-0352, Japan

## Abstract

Greater amberjack (*Seriola dumerili*) is distributed in tropical and temperate waters worldwide and is an important aquaculture fish. We carried out de novo sequencing of the greater amberjack genome to construct a reference genome sequence to identify single nucleotide polymorphisms (SNPs) for breeding amberjack by marker-assisted or gene-assisted selection as well as to identify functional genes for biological traits. We obtained 200 times coverage and constructed a high-quality genome assembly using next generation sequencing technology. The assembled sequences were aligned onto a yellowtail (*Seriola quinqueradiata*) radiation hybrid (RH) physical map by sequence homology. A total of 215 of the longest amberjack sequences, with a total length of 622.8 Mbp (92% of the total length of the genome scaffolds), were lined up on the yellowtail RH map. We resequenced the whole genomes of 20 greater amberjacks and mapped the resulting sequences onto the reference genome sequence. About 186,000 nonredundant SNPs were successfully ordered on the reference genome. Further, we found differences in the genome structural variations between two greater amberjack populations using BreakDancer. We also analyzed the greater amberjack transcriptome and mapped the annotated sequences onto the reference genome sequence.

## 1. Introduction

With the improvements in next generation sequencing technologies in the past few years, many genome projects of aquaculture fishes have been reported, including Atlantic salmon (*Salmo salar*) [1], Atlantic cod (*Gadus morhua*) [2], rainbow trout (*Oncorhynchus mykiss*) [3], Japanese flounder (*Paralichthys olivaceus*) [4], half-smooth tongue sole (*Cynoglossus semilaevis*) [5], platyfish (*Xiphophorus maculatus*) [6], common carp (*Cyprinus carpio*) [7], and channel catfish (*Ictalurus punctatus*) [8], and molecular markers of the shared genomic loci among individuals have been obtained for genotype-phenotype linkage analysis. Chromosome-level assemblies or assembled genome sequences integrated with genetic maps are powerful tools that enable analyses of fish genetic breeding by marker-assisted or gene-assisted selection as well as help identify functional genes for biological traits. However, there are a limited number of fish species for which chromosome-level genome assemblies are available.

Single nucleotide polymorphisms (SNPs) in whole genomes are one of the most important genomic resources for studying population diversity, conservation genetics, and functional gene identification for biological traits [9–13]. To obtain molecular markers of the shared genomic loci among individuals, many technologies have been developed to probe whole-genome polymorphisms. These techniques have allowed the synthesis of DNA probes that can be used on SNP microarrays [14], making it possible to explore genome-wide SNPs in a high-throughput manner. However, the cost of array design and application obstructs their wider use in nonmodel species, especially for economically important organisms [15]. More importantly, microarray approaches cannot discover novel SNP loci for species without reference sequences [16]. The development of state-of-the-art next generation sequencing platforms has enabled scientists to scan small variants in genomes on an unprecedented scale [17, 18]. Multiplex library strategies have been used widely to further reduce the cost per sample [19]. However, cost is still one of the biggest challenges for whole-genome resequencing in nonmodel organisms [20]. We are developing whole-genomic analysis of *Seriola* species to study how much genetic variation remains in natural fishes and to investigate the mechanism of whole genome duplication, as well as to obtain molecular markers of the shared genomic loci among individuals for genotype-phenotype linkage analysis and to identify functional genes for biological traits. We have reported radiation hybrid (RH) physical and linkage maps of yellowtail (*Seriola quinqueradiata*) [21, 22] and compared the synteny among four model fishes, because yellowtail is one of the most important fishery resources in Japan. Greater amberjack *(Seriola dumerili)* is evolutionally closely related to yellowtail and is more widely distributed in tropical and temperate waters than yellowtail. We are now interested in how much commonality exists between the yellowtail and amberjack genomes.

One of the purposes of this study was to construct a platform for quantitative trait locus (QTL), marker-assisted selection [23, 24], and gene-assisted selection [25, 26] programs for greater amberjack breeding based on SNPs. Therefore, we carried out de novo sequencing of the greater amberjack genome (hereafter referred to as the reference genome) and detected SNPs genome-wide using next sequencing technology and high-quality genome assembly. We then resequenced 20 greater amberjack genomes and identified SNPs on the reference genome sequence and analyzed genome-wide structural variations in two greater amberjack populations. In addition, we analyzed the RNA sequences from 12 amberjack tissues (muscle, brain, eye, heart, liver, intestine, kidney, spleen, gonad, gill, fin, and bladder) and mapped the resulting sequences onto the reference genome sequence to assemble them.

## 2. Materials and Methods

### 2.1. Ethics Statement

In Japan, field permits are not required for greater amberjack. The Institute Animal Care and Use Committee of the National Research Institute of Aquaculture (IACUC-NRIA 27004) approved the fish handling, husbandry, and sampling methods used in this study. We sampled sperm from one anesthetized male amberjack that had been cultured in the aquarium of the Komame Station of the National Research Institute of Aquaculture (Kochi, Japan). We gathered blood from eight greater amberjacks cultured in Komame Station and 12 individual greater amberjacks in the marine crop of Owasebussan Co. Ltd. (Mie, Japan). For RNA sequencing, we sampled tissues from one anesthetized greater amberjack fished in the Pacific Ocean near Mie Prefecture, Japan.

### 2.2. Whole-Genome Sequencing

We extracted high molecular weight genomic DNA from the sperm of one male greater amberjack and checked the DNA quality by spectrophotometer and 2% agarose gel electrophoresis before library construction. DNA fractions of 170–300 bp (for libraries with 250 bp insert size), 450–550 bp (for libraries with 500 bp insert size), and 700–900 bp (for libraries with 800 bp insert size) were excised and eluted from the gel slices overnight at 4°C in 300 *μ*l of elution buffer (5 : 1 [vol/vol] LoTE buffer [3 mM Tris–HCl (pH 7.5), 0.2 mM EDTA] to 7.5 M ammonium acetate) and purified using a Spin-X filter tube (Fisher Scientific, Waltham, MA, USA) and ethanol precipitation. Genome libraries were prepared using a modified paired-end tag protocol supplied by Illumina (http://prodata.swmed.edu/LepDB/Protocol/illumina_Paired-End_Sample_Preparation_Guide.pdf#search=%27pairedend+tag+protocol+Illumina%27).

Mate pair (aka jumping) libraries were constructed using 4 mg of genomic DNA with the Illumina Nextera Mate Pair library construction protocol and reagent (FC-132-1001). We amplified the 2 kbp and 5 kbp inserts with 10 cycles of PCR and the 10 kbp and 20 kbp inserts with 15 cycles of PCR. The two fractions were pooled for mate pair (100 bp) sequencing using an Illumina HiSeq 2500 system.

For sequencing on the PacBio RSII long read platform (Pacific Biosciences, Melon Park, CA, USA), the genomic DNA was sheared to 10 to 20 kb using an ultrasonicator (Covaris Inc., Woburn, MA, USA) and converted to the proprietary SMRTbell™ library format using an RS DNA Template Preparation Kit (Pacific Biosciences). SMRTbell templates were subjected to standard single-molecule real-time (SMRT) sequencing using an engineered phi29 DNA polymerase on a PacBio RS II system according to the manufacturer's protocol. We sequenced 8 Gbp of the 10 kbp library by PacBio RS II (10 kb library) using a SMRT cell.

We removed the 3′-end adaptor sequences and mate pair annular junctional adaptor sequences from the Illumina sequencing data with Cutadapt (http://cutadapt.readthedocs.io/en/stable/guide.html) and removed low-quality reads and reads shorter than 20 bp with a flexible read trimming tool, Trimmomatic (version 0.32) [27]. We assembled the paired-end short read sequences using SOAPdenovo2 assembly software [28], mapped the resulting contigs to the mate pair read sequences, and closed gaps between contigs with Platanus [29]. Then, we converted the mate pair sequences to reverse complemental sequences using the Reverse Complement tool in the FASTX-Toolkit (http://hannonlab.cshl.edu/fastx_toolkit/). We assembled the resulting contigs again using SOAPdenovo2 and mapped the PacBio RSII read data to long scaffolds using PBJelly [30] to remove sequence gaps.

### 2.3. Alignment of the Amberjack Genome Assembly

We mapped the amberjack genome sequence onto the yellowtail RH physical map [22]. Then, we used the sequences of the mapped markers of yellowtail for BLAST searches against the scaffolds in the amberjack genome assembly using BWA software [31]. When a query sequence had multiple hits, we selected the top hit for analysis only if its *E* value was less than half that of the second hit; all other hits were removed from further analysis. The amberjack genome assembly sequences were lined up on the yellowtail RH physical map based on the results of the BLAST searches.

### 2.4. Resequencing of 20 Greater Amberjack Genomes and SNP Detection

We resequenced high molecular weight genomic DNA extracted from the blood of 20 greater amberjacks. The DNA quality was checked by spectrophotometer and 2% agarose gel electrophoresis before library construction. DNA fractions of 200–300 bp (for libraries with 250 bp insert size) were excised and eluted from the gel slices and purified using a Spin-X Filter Tube (Fisher Scientific). Genome libraries were prepared using a modified paired-end tag protocol supplied by Illumina. We sequenced 20 Gbp of each library using an Illumina HiSeq 2500 system. We removed the adaptor sequences from both ends of the sequences with Cutadapt and trimmed the sequences using Trimmomatic (version 0.32) [27] to remove areas where the average CV (coefficient variation) was <20. We selected sequences that were >70 bp long and mapped them onto the amberjack reference genome sequence using the BWA software [31] to detect mutations. The sequences in regions where mismatches were high were realigned using the GATK software (Genome analysis tool kit, https://www.intel.co.jp/content/www/jp/ja/healthcare-it/solutions/genomicscode-gatk.html) to improve mapping accuracy.

### 2.5. Detection of Structural Variants by BreakDancer

We used BreakDancer-1.1 under GPLv3 [32], which provides genome-wide detection of structural variants from next generation paired-end sequencing reads, with the default parameters to detect insertions/deletions, inversion, and translocations in the pair-end resequenced data of the 20 greater amberjacks. The whole-genome resequenced assembly files were saved in Bam file format. These files were analyzed by BreakDancer to detect structural variations in each resequenced genome. In addition, we combined the resequenced data of the eight greater amberjacks captured off the Kochi Coast and combined the resequenced of the 12 greater amberjacks captured off Chinese Hainan Islands Coast and analyzed each of these data sets by BreakDancer. The structural variation data files were merged in a Circus plot (http://circos.ca/intro/genomic_data/) to visualize the structural variations on the genomes.

### 2.6. Transcriptome Sequencing

We bought a single alive female adult amberjack that was fished in the Pacific Ocean near Mie Prefecture for tissue sampling. Total RNAs were extracted from 12 tissues (muscle, brain, eye, heart, liver, intestine, kidney, spleen, gonad, gill, fin, and bladder) using RNAiso Plus (Takara, Shiga, Japan). After purification of poly(A) + RNA, first-strand cDNA synthesis was primed with an N6 randomized primer using a ScriptSeq RNA-Seq Library Prep Kit (Illumina). After hydroxyl apatite chromatography, the single-stranded cDNA was amplified by seven cycles of PCR. One sequencing run was performed on an Illumina HiSeq 2500 paired-end sequence platform using Illumina reagents and protocols. We removed the 3′ adaptor sequences from the read sequences with Cutadapt (http://cutadapt.readthedocs.io/en/stable/guide.html) and trimmed the read sequences using Trimmomatic (version 0.32) [27]. We mapped the trimmed sequences onto the amberjack reference genome using cufflinks (http://cole-trapnell-lab.github.io/cufflinks/) to identify transcribed regions of the genome. We then carried out BLASTX homology searches against the amino acid sequence data in the NCBI RefSeq Vertebrate Other dataset using Blast2GO [55].

## 3. Results and Discussion

### 3.1. Whole-Genome Sequence of Greater Amberjack

We prepared genomic DNA from the sperm of one male greater amberjack that had been cultured in the aquarium at the Komame Station of the National Research Institute of Aquaculture (Kochi, Japan). We sequenced three paired-end libraries and four mate pair libraries on an Illumina HiSeq 2500 platform (Supplementary Figure S1) and obtained 133.1 Gbp of sequence data with 200-fold coverage. Using the short-read de nova assembler SOAPdenovo2 [28], we obtained a greater amberjack genome assembly that was 663 Mbp long, with 724 scaffolds (>2 kbp) and N50 of 4.99 Mbp (Table 1). This high-quality assembly was obtained because the terminal sequences in the mate pair library integrated DNA fragments longer than 20 kbp, which was useful in building a more complete genome. We also sequenced 10 kbp libraries on a PacBio RSII platform with 10-fold coverage and obtained 7.27 Gbp of polymerase reads and 7.26 Gbp of subreads. Then, we mapped the assembled PacBio RSII sequence data to the assembled HiSeq 2500 sequence data, which allowed us to reduce the number of gaps in the scaffolds by one-third (Table 1). The final genome assembly was 678 Mbp long, with N50 of 5.8 Mbp (Table 1; DDBJ: BDQW01000001–BDQW01034655).

The Atlantic salmon (*Salmo salar*) reference genome assembly is 2.97 Gbp long with scaffold N50 of 2.97 Mbp [33]. For the common carp (*Cyprinus carpio*) reference genome assembly, the scaffold N50 is 1.0 Mbp [7]; for the channel catfish (*Ictalurus punctatus*) reference genome assembly, it is 7.73 Mbp [34]; for zebrafish (*Danio rerio*), it is 1.55 Mbp [35]; for stickleback (*Gasterosteus aculeatus*), it is 10.8 Mbp [36]; and for medaka (*Oryzias latipes*), it is 5.1 Mbp [37]. Therefore, the quality of the greater amberjack reference genome assembly is equal to or better than that of the other fish reference genome assemblies that have been published so far.

### 3.2. Alignment of the Assembled Amberjack Genome Sequence to the Yellowtail Radiation Hybrid Physical Map

**Figure 1:**
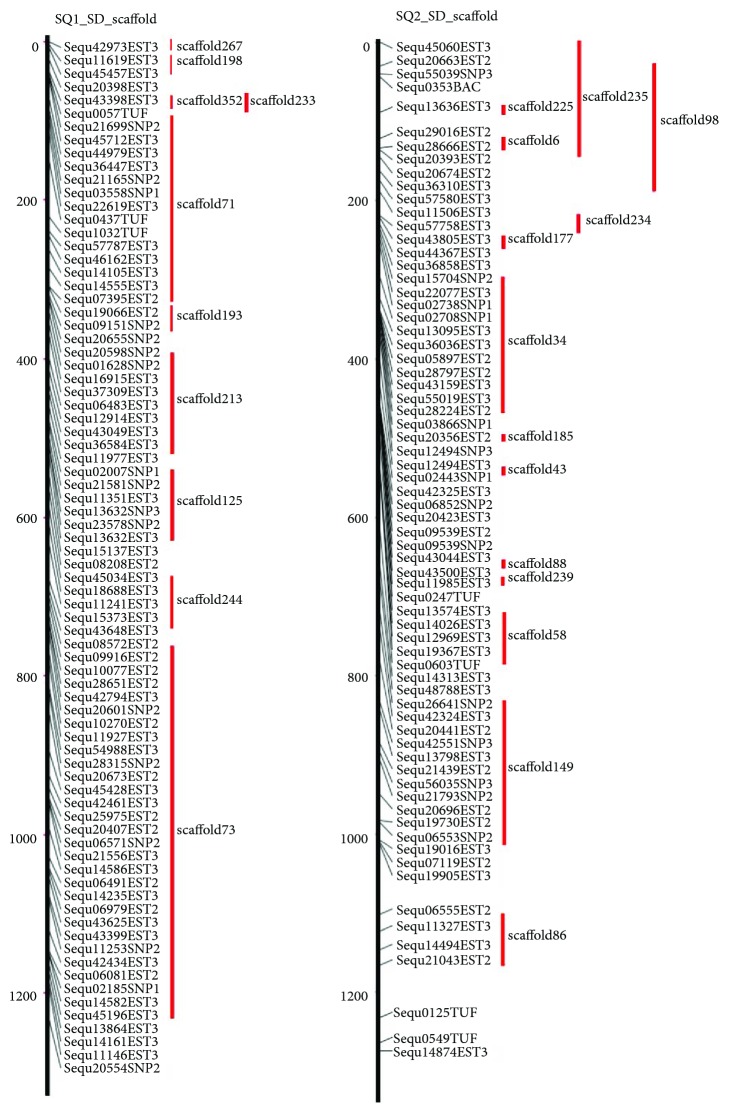
Greater amberjack scaffolds aligned onto two linkage groups of the yellowtail radiation hybrid physical map. A representative part of the yellowtail radiation hybrid (RH) physical map is shown with the greater amberjack scaffolds aligned. Numbers on the left indicate distance (cR) from the top of the RH map. Black lines indicate chromosomes. Red lines on the left indicate scaffold lengths. Seq numbers indicate mapped sequence number. Scaffold numbers identify the aligned scaffolds.

Linkage maps indicate the genetic distances of mapped genes, whereas physical maps, where the distance between mapped genes reflects their physical distance on a genome, are more advantageous to accurately align scaffolds. However, to produce a physical map of greater amberjack using radiation hybrid (RH) panels will take a long time, because greater amberjack epidermal cells take 72 hours to divide, and a lot of time is needed to build greater amberjack RH panels. In a previous study [22], we reported RH physical and linkage maps of yellowtail (*Seriola quinqueradiata*) and mapped 300 to 600 bp long expressed sequence tags (ESTs) onto the yellowtail RH map. Greater amberjack is closely related to yellowtail evolutionally, so we assumed that the genome sequences of greater amberjack and yellowtail will be highly homologous. We tried to map the assembled amberjack genome sequence onto the yellowtail RH map by BLAST searches using BWA software [31]. Then, we used the yellowtail ESTs for BLAST searches against the scaffolds in the amberjack genome assembly. As a result, 215 of the longest amberjack sequences (total length 622.8 Mbp making up 92% of the total length of the genome scaffolds) were mapped onto the yellowtail RH map (Figure 1, Table 2, Supplementary Figure S2). Therefore, we considered that the yellowtail RH physical map was useful for lining up the assembled reference genome sequences of greater amberjack. This result suggested that the chromosome construction of greater amberjack and yellowtail may be conserved.

### 3.3. Resequencing of Amberjack Genomes and SNP Detection

We resequenced 20 amberjack genomes on an Illumina HiSeq 2500 platform and obtained an average of 18 Gbp of sequence data with about 27-fold coverage for each genome. The proportion of high-quality bases (≥Q30) to the total number of bases in each genome sequence was an average of 91%, and mean quality scores of the reads was an average of 38 (Supplementary Table S1). We mapped 99% of the resequenced data onto the amberjack reference genome using BWA software (Supplementary Table S2), which resulted in 99.9% total coverage of the reference genome (Supplementary Table S3). We detected 7,059,071 mutations in the resequenced data mapped on the reference genome sequence by realigning the regions where mismatches were accumulated using Smatools and BCFtools (http://www.htslib.org/download/) and removing PCR duplicates using Picard (https://broadinstitute.github.io/picard/). We selected 186,259 nonredundant SNPs that showed polymorphisms at 10% or more in the 20 individuals and ordered them onto the reference genome sequence aligned on each linkage group (Table 3). There were 24 linkage groups corresponding to the 24 chromosomes.

Recently, SNP identification of Atlantic salmon, channel catfish, and common carp has been reported [11, 38, 39]. SNP discovery in Atlantic salmon was performed using extensive deep reduced representation sequencing, restriction site-associated DNA, and mRNA libraries derived from farmed and wild Atlantic salmon samples, resulting in the discovery of >400 K putative SNPs, 132,023 of which were selected for an Affymetrix Axiom SNP array [38]. SNP identification in the common carp genome was performed separately for three strains by resequence, and a total of 24,272,905 nonredundant SNPs were detected, and 223,274 of them were selected for a carp SNP array system [39]. SNP identification in the catfish genome was performed within five strains by resequence, and 237,655 significant SNPs were detected and assigned to 29 tentative chromosomes based on the catfish linkage map [40]. Putative 2.12 million SNPs were identified in the rainbow trout genome within 12 fish by resequence, and 49,468 SNPs were finally selected for an Affymetrix SNP array [41]. We detected 7,059,071 mutations within the 20 greater amberjack genomes and selected 186,259 nonredundant SNPs for a SNP array. Thus, our SNP identification analysis of the greater amberjack genome produced SNP numbers that were about equal to those of the other aquaculture fish species.

To obtain molecular markers of shared genomic loci among individuals, many high-throughput technologies have been used to probe whole-genome polymorphisms, for example, SNP microarrays [10, 12], digital PCR [42], mass spectrometer SNP genotyping [43], and next generation sequencing [44]. The SNP information on the greater amberjack reference genome sequence that we obtained may be very useful for breeding amberjack by marker-assisted, gene-assisted, or genomic selection and for identifying functional genes for biological traits [45].

### 3.4. Detection of Structural Variants by BreakDancer

Recent developments in the analytical capacity of DNA sequencers have meant that massively parallel sequencing has been carried out in some species [46–48], making multiple data and methods available for the detection of structural variations. It has been suggested that small insertions/deletions and large structural variations may be major contributors to genetic diversity and traits [49–53].

**Figure 2:**
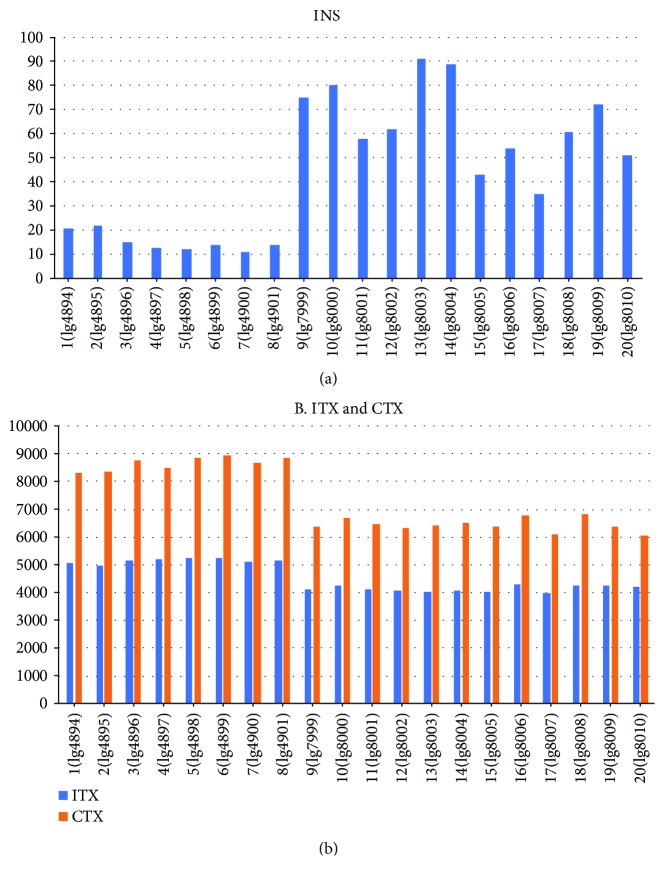
Comparison of structural variations in two greater amberjack populations. Greater amberjack samples 1–8 were captured in the sea near Kochi Prefecture, Japan. Greater amberjack samples 9–20 were captured in the sea near the Chinese Hainan Islands. (a) Number of insertions (INS) and (b) number of intra- (ITX) and inter- (CTX) chromosomal translocations detected in the 20 genomes. The vertical axis shows the number of structural variations.

**Figure 3:**
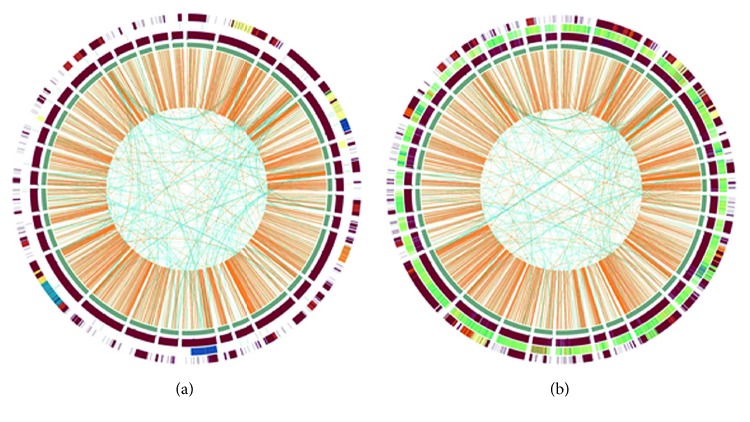
Genome-wide landscape of structural variations of greater amberjack. We linked the BreakDancer data to Circus plots to visualize regions of the genome that contained structural variations. We combined the resequenced data of (a) eight greater amberjacks captured off the Kochi coast and (b) 12 greater amberjacks captured off the Chinese Hainan Islands coast and analyzed these data by BreakDancer. Gene density of each contig is visualized by dark lines. The outermost circle shows inversion, next circle shows insertion, and third circle shows deletion. Orange lines show intrachromosomal translocations, and blue lines show interchromosomal translocations.

We carried out BreakDancer [32] analysis to detect structural variants in the greater amberjack genome using resequenced data of the 20 greater amberjacks mapped onto the reference genome sequences, and the results are shown in Figures 2 and 3 and Table 4. BreakDancer Max can predict five types of structural variants (insertions, deletions, inversions, and intra- and interchromosomal translocations) from next generation short paired-end sequencing reads using read pairs that are mapped with unexpected separation distances or orientations [32]. Greater amberjack samples 1–8 (Figure 2, Table 4) were captured off the sea of Kochi Prefecture, Japan (Pacific Ocean side of Japan) and cultured in the Komame Station, National Research Institute of Aquaculture, and samples 9–20 (Figure 2, Table 4) were captured off the sea around the Chinese Hainan Islands and cultivated in the marine crop of Owasebussan Co. Ltd. in Mie, Japan. We found an average of 19,083 structural variations per genome. The structural variations in the genomes of the Kochi amberjacks were different from those in the genomes of the Chinese amberjacks. More short insertions were detected in the Chinese amberjack genomes than in the Kochi amberjack genomes (Figure 2(a), Table 4), whereas more intra- and interchromosome translocations were detected in the Kochi amberjack genomes than in Chinese amberjack genomes (Figure 2(b), Table 4). The total number of structural variations (average 21,634) in the Kochi amberjack genomes were more than those (average 17,381) in the Chinese amberjack genomes. It is unclear why the greater amberjack captured off Kochi (Pacific Ocean side of Japan) had more chromosome translocations than the Chinese greater amberjack, and the greater amberjacks captured off China had many insertions in their genomes.

Greater amberjack is found in subtropical regions throughout the globe. In the Indo-West Pacific, this species has been reported from South Africa, the Persian Gulf, southern Japan, and the Hawaiian Islands, south to New Caledonia, and the Mariana and Caroline Islands in Micronesia. In the western Atlantic Ocean, greater amberjack is found off Nova Scotia, Canada south to Brazil including Bermuda, the Gulf of Mexico, and the Caribbean Sea. Gold and Richardson [54] analyzed the population structures of greater amberjack from the Gulf of Mexico and from the western Atlantic Ocean using variations of mitochondria DNA and suggested that one subpopulation existed in the northern Gulf of Mexico and a second subpopulation existed along the southeast Atlantic coast of America. Similarly, the results of our structural variation analysis may demonstrate that greater amberjack captured off the Hainan Islands in China, and Kochi in Japan could be classified as two subpopulations. If this is the case, the genome structural variations in the Chinese and Kochi greater amberjack subpopulations may have developed in different ways depending on the environment or population size because Hainan Islands in the South China Sea and Kochi on the Pacific side of Japan are geographically separated.

We combined the resequenced data of the eight Kochi amberjacks and combined the resequence data of the 12 Chinese amberjacks and analyzed separately their structure variations using BreakDancer. Then, we analyzed the distribution of the structural variations in the amberjack genomes using a Circus plot linked with the data obtained using BreakDancer (Figure 3). We found that the short insertions occurred genome wide in the Chinese amberjack (Figure 3(b)) and intra- and interchromosomal translocations occurred genome wide between narrow regions in the Kochi and Chinese amberjack populations (Figures 3(a) and 3(b)). There were some chromosome areas where intrachromosome translocations occurred in some individuals and interchromosome translocations occurred in other individuals (Figure 3(a), 3(b)).

This study is the first to analyze genome structural variations in fish species. However, we analyzed only 8 and 12 individuals in two populations. In future studies, we plan to resequence the genomes of more individuals to detect more accurately whole-genome structural variations in the greater amberjack genome.

### 3.5. Transcriptome Analysis

**Figure 4:**
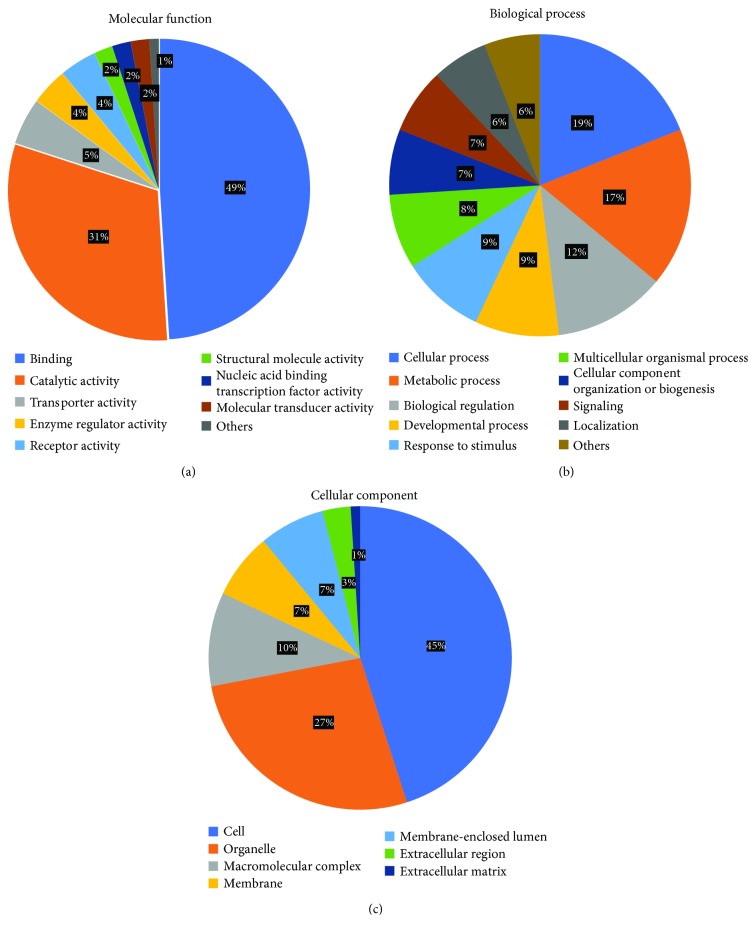
Gene ontology terms assigned to the assembled cDNA transcripts of greater amberjack. The gene ontology annotations under the three main categories: (a) molecular function, (b) biological process, and (c) cellular component.

We analyzed the RNA sequences from 12 greater amberjack tissues (muscle, brain, eye, heart, liver, intestine, kidney, spleen, gonad, gill, fin, and bladder) by Illumina HiSeq 2500 sequencing. The trimmed reads were assembled by Cufflinks using the amberjack reference genome with BWA software [31]. We successfully mapped all the RNA sequences onto the reference genome sequence. After assembly, we obtained a total of 45,109 transcripts with N50 of 2624 bp; the longest contig was 13,141 bp, and the average contig length was 1241 bp. BLASTX searches (*E* value <1e − 5) were performed against the RefSeq vertebrate other sequences in NCBI using Blast2GO [55]. We identified 35,456 transcripts that shared high homology with RefSeq sequences (DDBJ: IACO01000001–IACO01045109), and each transcript was assigned to at least one gene ontology (GO) term under one of the three main categories: biological process, molecular function, and cellular component. The majority of assigned GO terms were under molecular function (41%), followed by biological process (35%), and cellular component (24%) (Figure 4). Under molecular function, binding and catalytic activity represented about 80% of the GO terms (Figure 4(a)); under biological process, cellular and metabolic processes (36%) and biological regulation (12%) were highly represented (Figure 4(b)); and under cellular component, cell and organelle represented about 72% of the GO terms (Figure 4(c)).

In a previous study, we reported the transcriptome assembly and GO analysis of yellowtail [22]. The GO analyses of the amberjack and yellowtail transcripts were similar and also similar to the results obtain for other fish species [56–58].

## 4. Conclusions

We carried out de novo genome sequence analysis and resequencing of 20 greater amberjacks to detect SNPs and to order the SNPs in aligned scaffolds. We obtained a high-quality genome assembly of 678 Mbp with N50 of 5.8 Mbp, and 215 scaffolds with 187,00 SNPs were ordered using the yellowtail RH physical map and homology between the yellowtail and greater amberjack genome sequences. Further, we analyzed structural variations in greater amberjack genomes using resequence data and found differences in the structural variations between two populations. We also analyzed the greater amberjack transcriptome, mapped the annotated sequences onto the reference genome sequence, and identified 35,456 transcripts that shared high homology with RefSeq sequences.

## Figures and Tables

**Table 1:** Summary statistics of the whole-genome sequence assembly of greater amberjack.

	Hiseq 2500	Hiseq + PacBio
Total bases	662,587,481 bp	677,669,644 bp
Number of scaffolds	34,824	11,655
Number of gaps	32,742	9742
Mean of scaffolds	19,026 bp	19,554 bp
Longest bases	22,167,742 bp	24,919,768 bp
N50	4,989,656 bp	5,812,906 bp
Number of >2Kb	724	707
Total bases (>2Kb)	655,539,910 bp	670,698,073 bp

The HiSeq 2500 sequence assembly was compared with the HiSeq 2500 sequence data mapped onto the PacBio RSII sequence data.

**Table 2:** Number of mapped scaffolds and total length of the scaffolds of greater amberjack mapped to the 24 linkage groups onto yellowtail RH physical map.

LGNo	The number of mapped scaffold	Total length (bp) of mapped scaffolds on each LG (bp)
1	10	32,041,590
2	13	28,988,571
3	9	30,680,262
4	9	31,420,922
5	3	22,220,271
6	4	28,673,657
7	2	23,029,943
8	9	27,651,179
9	6	35,439,400
10	9	26,409,569
11	6	10,977,986
12	12	25,517,532
13	9	31,109,094
14	14	21,191,548
15	9	26,971,016
16	12	25,150,760
17	20	19,289,833
18	6	26,805,652
19	6	26,928,810
20	4	28,110,268
21	12	23,144,198
22	15	15,779,806
23	2	25,211,550
24	14	30,054,902
Total	215	622,798,319

LGNo indicates the linkage group number; number of mapped scaffold indicates the number of scaffolds mapped onto each linkage group; and total length of mapped scaffolds on each LG indicates the total length (bp) of the scaffold sequences mapped onto each linkage group.

**Table 3:** Summary of nonredundant SNPs mapped to the 24 linkage groups by resequencing 20 greater amberjack genomes.

LGNo	The number of mutations in each LG	The number of mapped SNPs onto each LG
1	373,859	7831
2	375,132	7968
3	360,266	7933
4	334,990	7725
5	269,110	7413
6	311,326	7640
7	262,037	7763
8	309,899	7802
9	383,874	7980
10	310,587	7791
11	132,260	7172
12	285,525	7602
13	323,954	7722
14	227,417	7583
15	259,349	7763
16	267,369	7882
17	253,519	7833
18	280,457	7785
19	300,795	7878
20	312,122	7870
21	273,678	7831
22	220,279	7863
23	300,003	7800
24	331,264	7829
Total	7,059,071	186,259

LGNo indicates the linkage group number; the number of mutations in each LG indicates the number of mutations found in each linkage group; and the number of mapped SNPs onto linkage group indicates the number of SNPs ordered onto each linkage group.

**Table 4:** Structural variations detected in the genomes of 20 greater amberjack.

Sample number	DEL	INS	INV	ITX	CTX
1(Ig4894)	6921	21	737	5042	8293
2(Ig4895)	7447	22	730	4954	8346
3(Ig4896)	6458	15	760	5147	8745
4(Ig4897)	7690	13	731	5213	8504
5(Ig4898)	6821	12	716	5262	8852
6(Ig4899)	7146	14	742	5243	8946
7(Ig4900)	7383	11	771	5118	8672
8(Ig4901)	6789	14	751	5159	8863
9(Ig7999)	5948	75	653	4107	6347
10(Ig8000)	6157	80	693	4236	6699
11(Ig8001)	6085	58	636	4133	6456
12(Ig8002)	5989	62	665	4061	6343
13(Ig8003)	6015	91	657	4027	6403
14(Ig8004)	6112	89	701	4056	6503
15(Ig8005)	5879	43	654	4025	6370
16(Ig8006)	6269	54	680	4290	6759
17(Ig8007)	6034	35	661	3990	6108
18(Ig8008)	6180	61	677	4261	6822
19(Ig8009)	5974	72	686	4230	6351
20(Ig8010)	6300	51	650	4201	6068

Structural variations were detected by BreakDancer using pair-end resequenced data for 20 greater amberjack genomes. Sample numbers 1–8 represent greater amberjacks captured near the Kochi coast, and sample numbers 9–20 represent greater amberjacks captured near Chinese Hainan Islands. The Ig numbers are the resequenced data analysis numbers. DEL: deletion; INS: insertion; INV: inversion; ITX: intrachromosomal translocation; and CTX: interchromosomal translocation.

## Data Availability

The greater amberjack (*Seriola dumerili*) genome and transcriptome sequences have been deposited in the DNA Data Bank of Japan (DDBJ) under accession numbers BDQW01000001–BDQW01034655 (Biosample ID: SAMD00083043_ sdu_WGS.acclist.zip) and IACO01000001–IACO01045109 (Biosample ID: SAMD00084266 _sduTSA.acclist.txt), respectively.

## Supplementary Materials

Supplementary 1 Figure S1: distribution of the lengths of the short read sequences obtained by HiSeq 2500 sequencing. Paired-end and mate pair libraries was constructed and sequenced using an Illumina HiSeq 2500 system. The vertical axis shows the percentage of each read length, and the horizontal axis shows the sequence length.

Supplementary 2 Figure S2: greater amberjack scaffolds aligned onto the linkage groups of the yellowtail radiation hybrid physical map. The yellowtail radiation hybrid (RH) physical map is shown with the greater amberjack scaffolds aligned. Numbers on the left indicate distance (cR) from the top of the RH map. Black line indicates chromosomes. Red lines on the left indicate scaffold lengths. Seq numbers indicate mapped sequence number. Scaffold numbers identify the aligned scaffold.

Supplementary 3 Table S1: summary of the quality of the 20 amberjack resequenced genomes. The 20 greater amberjack genomes were resequenced in parallel, and an average of 18 Gbp was obtained for each genome.

Supplementary 4 Table S2: summary of the results of mapping the resequenced reads on the greater amberjack reference genome. Overall, 99% of the resequenced reads in all 20 genomes were successfully mapped onto the reference genome sequence.

Supplementary 5 Table S3: summary of sequence coverage information. The coverage information was obtained by filtering the resequencing data and mapping it onto the greater amberjack reference genome sequence.
