# Supplementary material for: Whole Genome Sequencing of Greater Amberjack (Seriola dumerili) for SNP Identification on Aligned Scaffolds and Genome Structural Variation Analysis Using Parallel Resequencing
Source: Int J Genomics. 2018 Mar 28;2018:7984292. doi: 10.1155/2018/7984292 (PMC5896239; doi:10.1155/2018/7984292)
Supplement: Supplementary 1 — Figure S1: distribution of the lengths of the short read sequences obtained by HiSeq 2500 sequencing. Paired-end and mate pair libraries was constructed and sequenced using an Illumina HiSeq 2500 system. The vertical axis shows the percentage of each read length, and the horizontal axis shows the sequence length. [file 7984292.f1.pptx]

## Slide 1
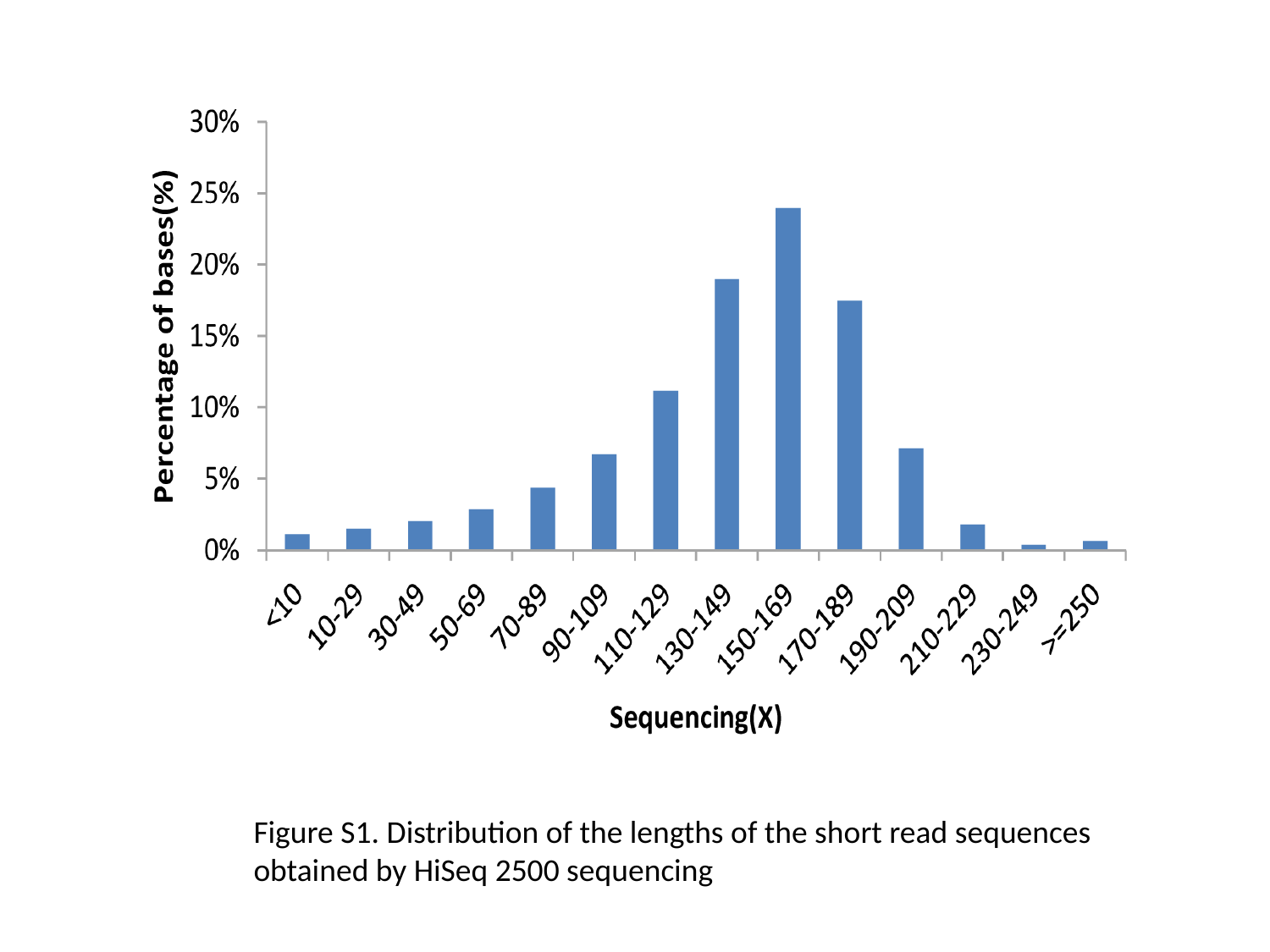

Figure S1. Distribution of the lengths of the short read sequences obtained by HiSeq 2500 sequencing
