# Supplementary material for: Whole Genome Sequencing of Greater Amberjack (Seriola dumerili) for SNP Identification on Aligned Scaffolds and Genome Structural Variation Analysis Using Parallel Resequencing
Source: Int J Genomics. 2018 Mar 28;2018:7984292. doi: 10.1155/2018/7984292 (PMC5896239; doi:10.1155/2018/7984292)
Supplement: Supplementary 2 — Figure S2: greater amberjack scaffolds aligned onto the linkage groups of the yellowtail radiation hybrid physical map. The yellowtail radiation hybrid (RH) physical map is shown with the greater amberjack scaffolds aligned. Numbers on the left indicate distance (cR) from the top of the RH map. Black line indicates chromosomes. Red lines on the left indicate scaffold lengths. Seq numbers indicate mapped sequence number. Scaffold numbers identify the aligned scaffold. [file 7984292.f2.pptx]

## Slide 1
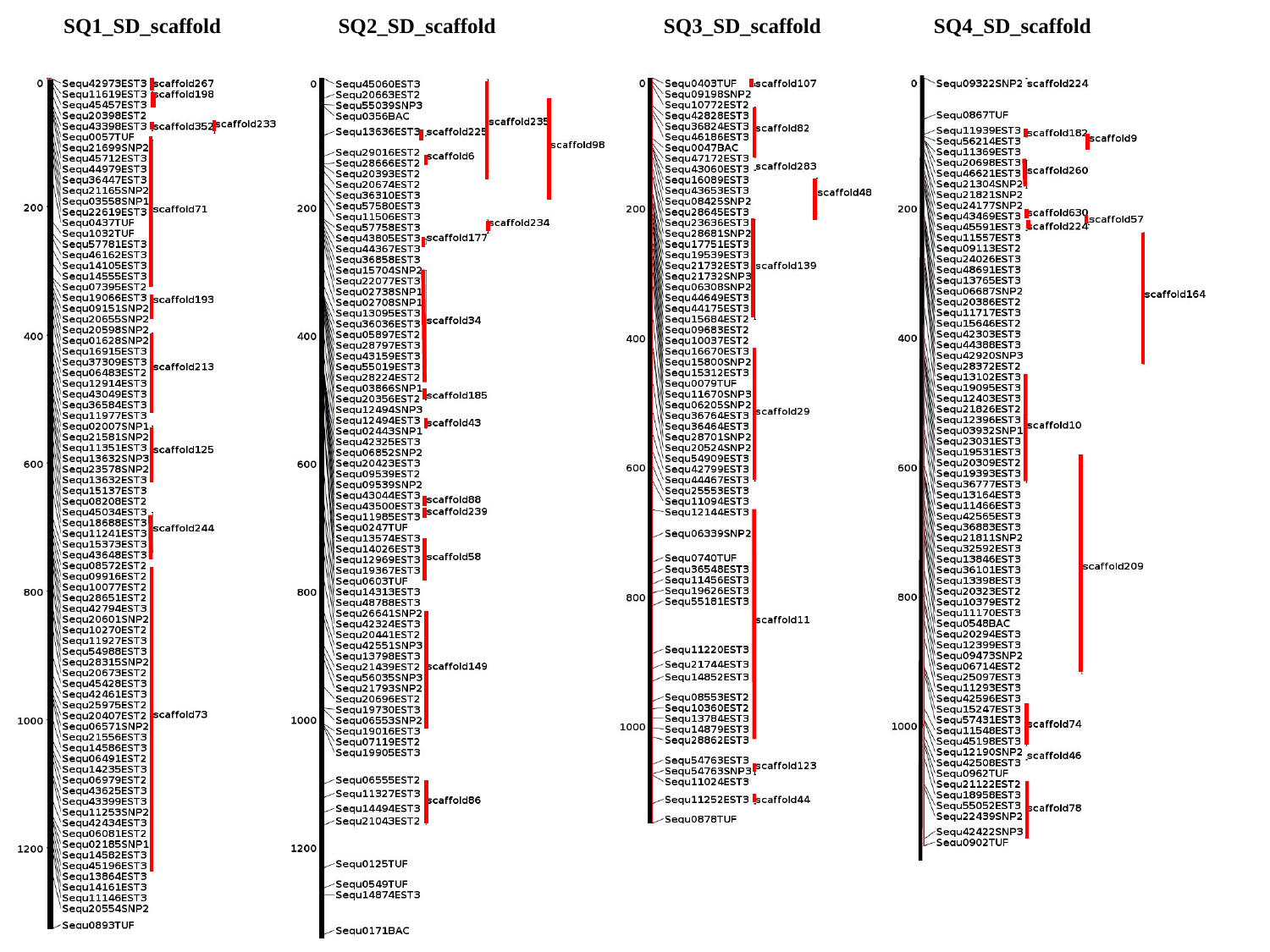

SQ1_SD_scaffold
SQ2_SD_scaffold
SQ3_SD_scaffold
SQ4_SD_scaffold

## Slide 2
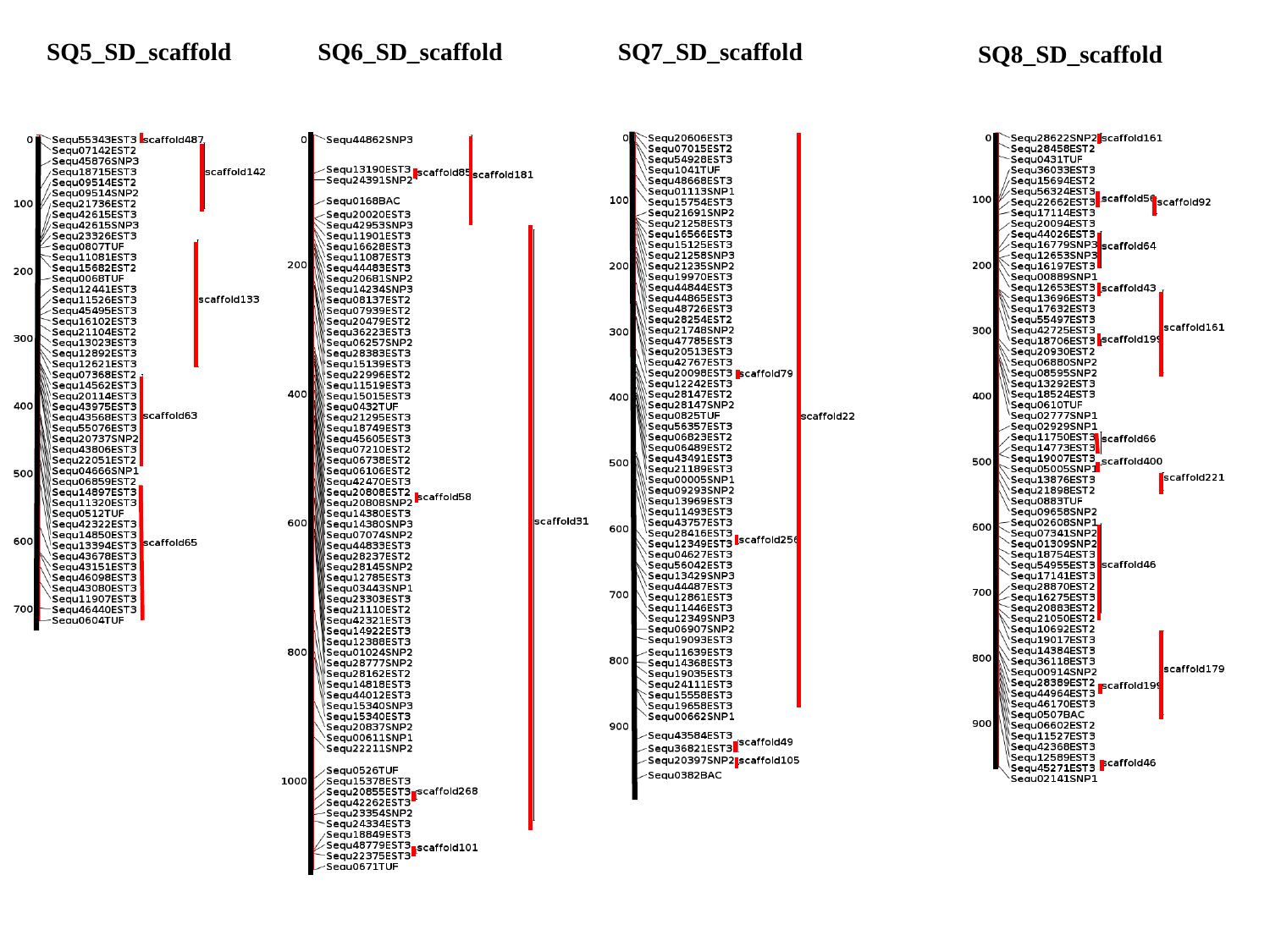

SQ5_SD_scaffold
SQ6_SD_scaffold
SQ7_SD_scaffold
SQ8_SD_scaffold

## Slide 3
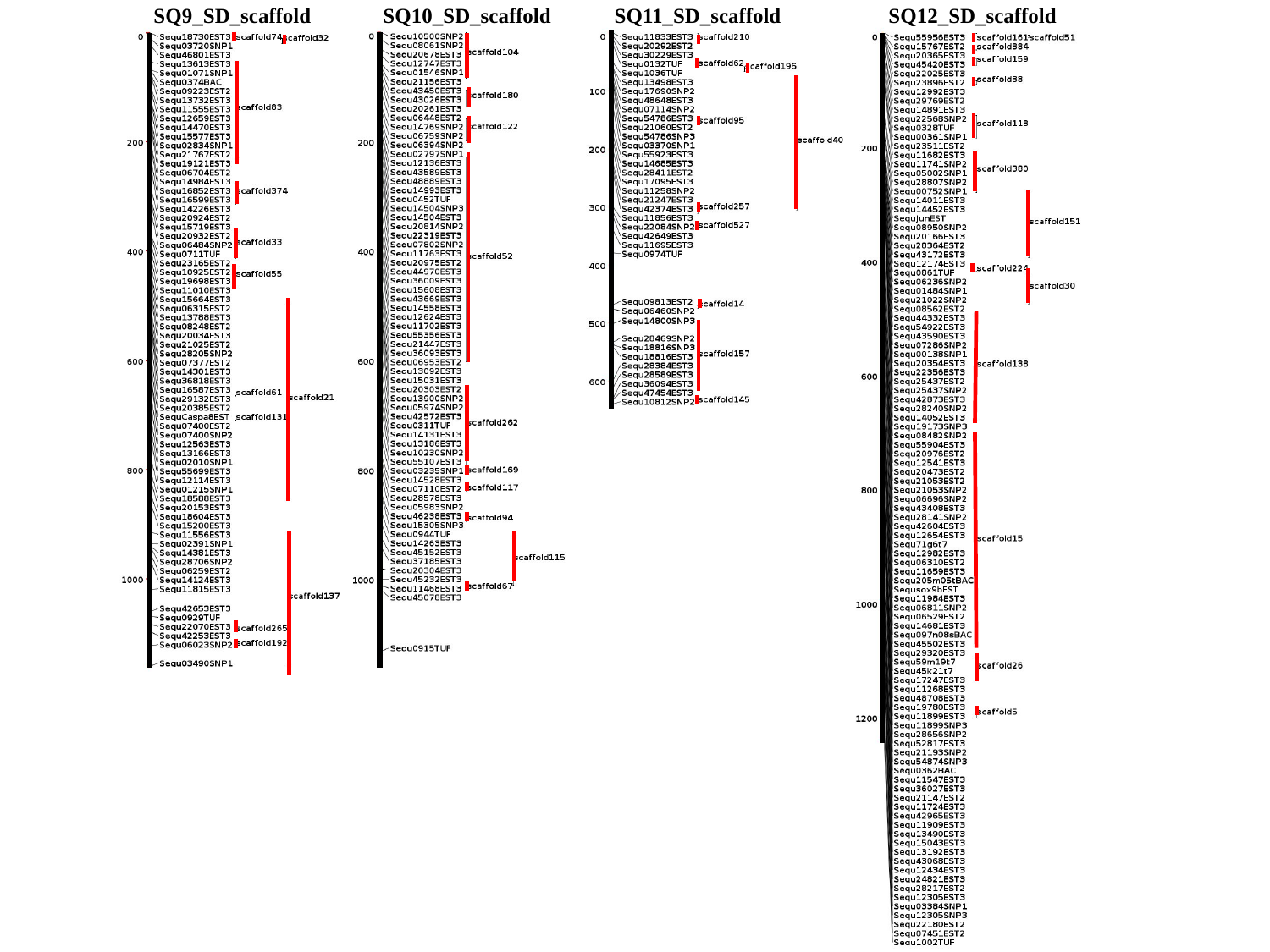

SQ9_SD_scaffold
SQ10_SD_scaffold
SQ11_SD_scaffold
SQ12_SD_scaffold

## Slide 4
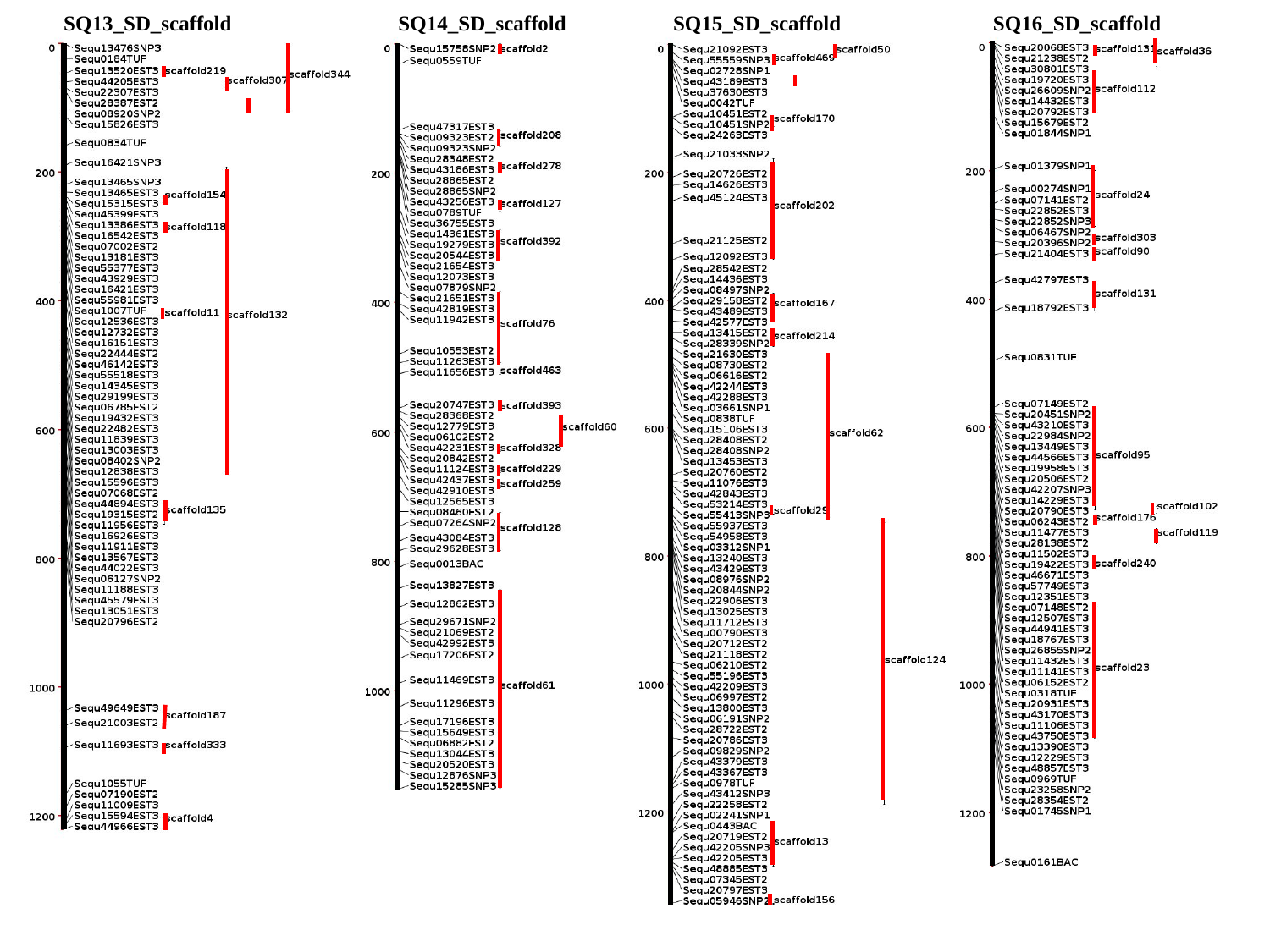

SQ13_SD_scaffold
SQ14_SD_scaffold
SQ15_SD_scaffold
SQ16_SD_scaffold

## Slide 5
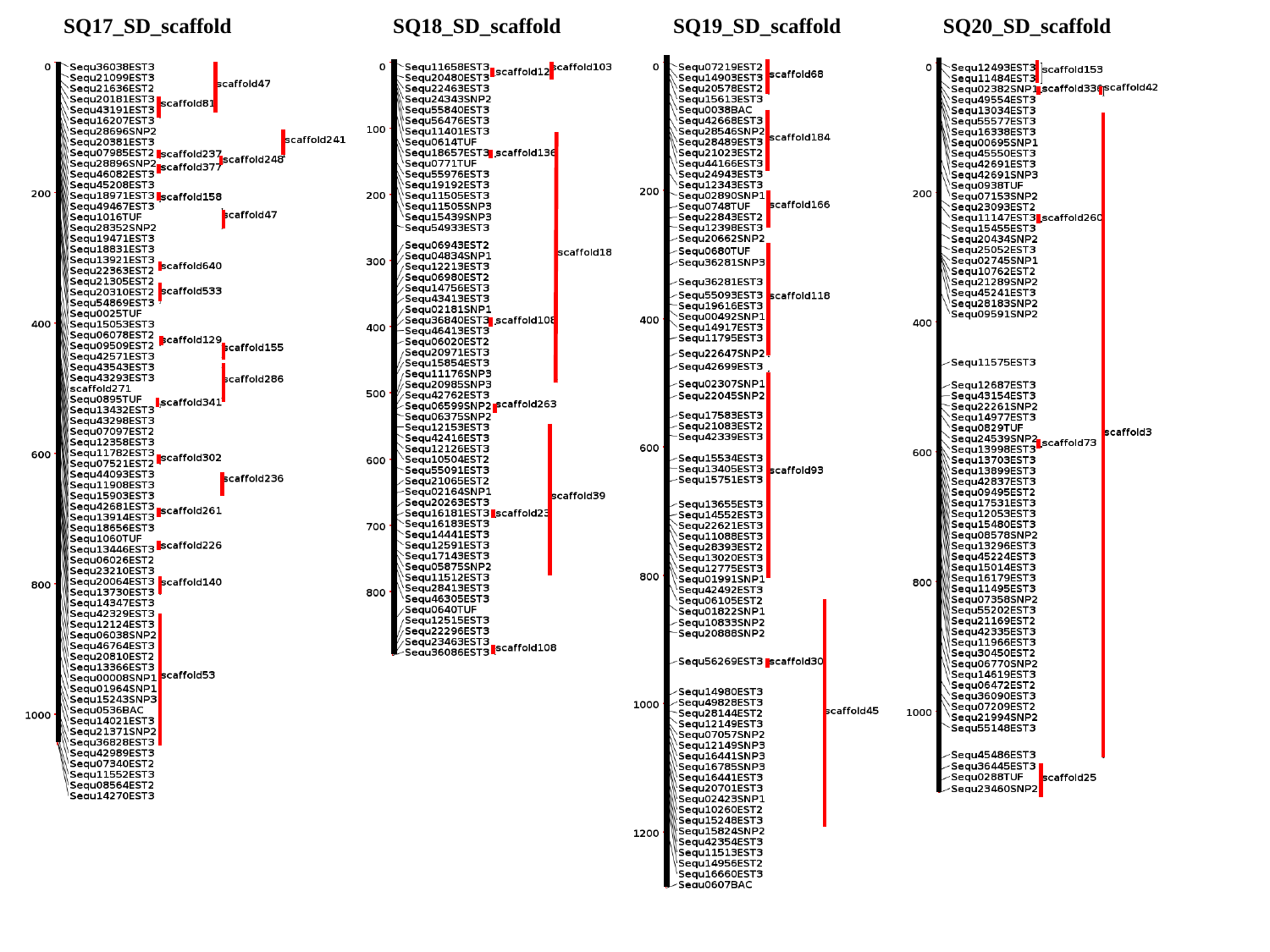

SQ17_SD_scaffold
SQ18_SD_scaffold
SQ19_SD_scaffold
SQ20_SD_scaffold

## Slide 6
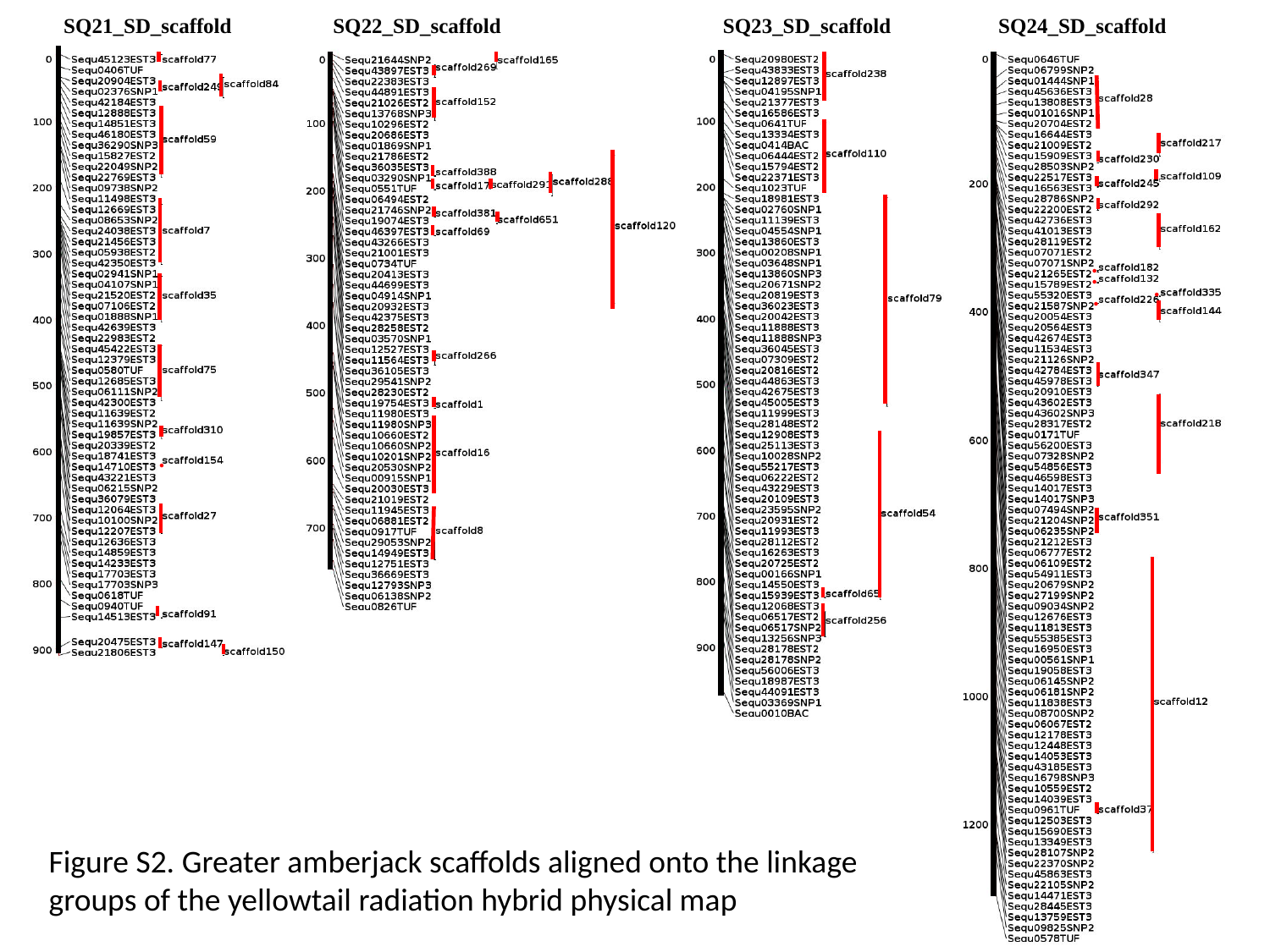

SQ21_SD_scaffold
SQ22_SD_scaffold
SQ23_SD_scaffold
SQ24_SD_scaffold
.
.
.
.
.
Figure S2. Greater amberjack scaffolds aligned onto the linkage groups of the yellowtail radiation hybrid physical map
